# Relationship of vitamin D status and bone mass according to vitamin D-binding protein genotypes

**DOI:** 10.1186/s12937-015-0016-1

**Published:** 2015-03-24

**Authors:** Hataikarn Nimitphong, Chanika Sritara, La-or Chailurkit, Suwannee Chanprasertyothin, Wipa Ratanachaiwong, Piyamitr Sritara, Boonsong Ongphiphadhanakul

**Affiliations:** 1 Department of Medicine and Research Center, Faculty of Medicine, Ramathibodi Hospital, Mahidol University, Rama VI Road, Ratchathewi, Bangkok 10400 Thailand; 2 Department of Radiology, Ramathibodi Hospital, Mahidol University, Rama VI Road, Ratchathewi, Bangkok 10400 Thailand; 3 Health Office, Electricity Generating Authority of Thailand, Nonthaburi, 11130 Thailand

**Keywords:** BMD, Bone turnover markers, Fetuin-A, 25(OH)D, *DBP* rs2282679 genotypes

## Abstract

**Background:**

Vitamin D-binding protein (DBP) may alter the biological activity of total 25-hydroxyvitamin D [25(OH)D]; this could influence on the effects of vitamin D in relation to bone mineral density (BMD) and fractures. Emerging data suggest that fetuin-A may be involved in bone metabolism. We aimed to investigate the influence of *DBP* gene polymorphism on the relationship of vitamin D status and fetuin-A levels to BMD and bone markers.

**Methods:**

This cross-sectional study was part of a health survey of employees of the Electricity Generating Authority of Thailand (1,734 healthy subjects, 72% male). Fasting blood samples were assayed for 25(OH)D, fetuin-A, N-terminal propeptides of type 1 procollagen (P1NP), C-terminal cross-linking telopeptides of type I collagen (CTx-I), and *DBP* rs2282679 genotypes. L1–L4 lumbar spine and femoral BMD were measured using dual-energy X-ray absorptiometry.

**Results:**

The *DBP* rs2282679 genotype distribution conformed to the Hardy–Weinberg equilibrium. There were no correlations between 25(OH)D levels and BMD and bone markers. But a trend of positive correlation was observed for the *DBP* genotypes with total hip BMD, and for the interaction between 25(OH)D and *DBP* genotypes with BMD at all femoral sites. We further analyzed data according to *DBP* genotypes. Only in subjects with the AA (common) genotype, 25(OH)D levels were positively related to BMD and bone markers, while fetuin-A was negatively related to total hip BMD, independently of age, gender and BMI.

**Conclusions:**

The interaction between vitamin D status, as measured by circulating 25(OH)D and *DBP* rs2282679 genotypes, modified the association between 25(OH)D and BMD and bone markers. Differences in *DBP* genotypes additionally influenced the correlation of fetuin-A levels with femoral BMD.

**Electronic supplementary material:**

The online version of this article (doi:10.1186/s12937-015-0016-1) contains supplementary material, which is available to authorized users.

## Background

Vitamin D plays important roles in bone and calcium metabolism. It enhances intestinal calcium absorption and suppresses bone resorption through its negative regulatory influence on parathyroid hormone secretion [1]. Moreover, vitamin D affects osteoblast by inhibiting proliferation but promoting mineralization and maturation [2,3]. Osteomalacia is a clinical feature of severe vitamin D deficiency due to impaired bone mineralization [4]. The influence of vitamin D on bone mass and the propensity to osteoporosis is less clear. Despite its biological effects related to bone mass, results from clinical studies investigating the effects of vitamin D on osteoporosis or osteoporotic fractures have been inconsistent [5,6]. Observational studies regarding the effect of vitamin D are usually performed using circulating 25-hydroxyvitamin D [25(OH)D], which is mostly bound to vitamin D-binding protein (DBP). It has been shown that genetic polymorphisms of *DBP,* for example three major polymorphic forms of *DBP*: GC1F, GC1S and GC2 are highly associated with 25(OH)D levels[7,8]. Recently, large genome-wide association studies in European [9,10] and two studies in Chinese [11,12] reported another *DBP* polymorphism, rs2282679, had an association with vitamin D deficiency. Nonetheless, data of the relationship between *DBP* rs2282679 genotypes and BMD and bone markers is scanty. It is unclear if there is an interaction of DBP or *DBP* genetic polymorphism and circulating 25(OH)D that affects bone mass; this may underlie the inconsistent results of some studies.

Fetuin-A is a multifunctional protein of hepatic origin. Besides glucose and energy homeostasis [13], fetuin-A may be involved in bone metabolism, as suggested by recent findings in elderly men and women [14,15]. With regard to the influence of vitamin D, it has been shown that vitamin D administration increase circulating fetuin-A in both experimental animals [16] and humans [17]. However, the relative influence of fetuin-A versus vitamin D and their possible interaction on bone mass is unknown at present. Therefore, the purpose of the present study was to investigate the influence of the interrelationship of vitamin D status, *DBP* gene polymorphism and fetuin-A levels on bone mineral density (BMD).

## Methods

This study was part of a health survey of 1,734 employees of the Electricity Generating Authority of Thailand (EGAT). Prior to commencement, the study was approved by the Committee on Human Rights Related to Research Involving Human Subjects, Faculty of Medicine, Ramathibodi Hospital, Mahidol University; all subjects gave written informed consent. As described in detail elsewhere [18], survey data was collected through self-administered questionnaires, physical examinations, electrocardiography, chest radiography, and blood analysis. Anthropometric variables, including weight, height and waist circumference (WC), were measured using standard techniques. Body mass index (BMI) was derived by weight (kg)/height (m)^2^. Fasting blood samples were obtained and assayed for 25(OH)D, fetuin-A, N-terminal propeptides of type 1 procollagen (P1NP), C-terminal cross-linking telopeptides of type I collagen (CTx-I), and *DBP* rs2282679 genotypes.

### BMD

The measurement method was described in an earlier report [19]. Each subject changed into light clothing before undergoing BMD assessment by dual-energy X-ray absorptiometry (DXA) at the lumbar spine (L1–L4 vertebrae) and total hip. All procedures were performed according to the recommendations of the International Society for Clinical Densitometry (ISCD) [20] by ISCD-certified technologists using a Hologic QDR-4500 DXA scanner (Bedford MA, USA). Quality assurance procedures using a spine phantom were performed daily. The precision error was less than 1.5%. The BMD coefficients of variation were 0.82% and 1.51% for the lumbar spine and total hip, respectively.

### *DBP* rs2282679 genotypes

Genomic DNA was isolated from peripheral blood leukocytes using a standard phenol–chloroform extraction method. The *DBP* rs2282679 polymorphism on chromosome 4q12-q13 was genotyped using a TaqMan® assay with allele-specific probes on an ABI Prism® 7500 Real-Time PCR System (Applied Biosystems, Foster City CA, USA). These polymorphisms were chosen because of recently large genome-wide association studies in populations of European descent reported that this genotype had the strongest association with vitamin D deficiency [9,10] and a similar result was reported in two studies of Chinese people [11,12].

### Fetuin-A level

Serum fetuin-A level was measured by sandwich enzyme immunoassay (R&D Systems, Minneapolis MN, USA). Intra- and inter-assay precisions were 4.9% and 7.3%, respectively.

### Serum 25(OH)D measurement

Serum 25(OH)D_2_ and 25(OH)D_3_ were analyzed by LC-MS/MS with an Agilent 1200 Infinity liquid chromatograph (Agilent Technologies, Waldbronn, Germany) coupled to a QTRAP® 5500 tandem mass spectrometer (AB SCIEX, Framingham MA, USA) using a MassChrom® 25-OH-Vitamin D_3_/D_2_ diagnostics kit (ChromSystems, Gräfelfing, Germany). The summation of serum 25(OH)D_2_ and 25(OH)D_3_ [total 25(OH)D] was used to reflect vitamin D status. Vitamin D deficiency was defined as having 25(OH)D levels of less than 50 nmol/L [20 ng/mL] [21]. The inter-assay and intra-assay coefficients of variation of total serum 25(OH)D level were 6.3% and 5.0%, respectively.

### Serum P1NP and CTx-I levels

Serum P1NP and CTx-I levels were determined by electrochemiluminescence immunoassay on a Cobas e 411 analyzer (Roche Diagnostics, Mannheim, Germany). The assays had intra-assay precision of 5.4% and 3.8%, respectively.

### Statistical analysis

Data were expressed as mean ± standard deviation (SD). All data were normally distributed. Differences between males and females were assessed by Student’s *t*-test. Multiple linear regression analysis was performed to identify the association between lumbar spine L1–L4 BMD, femoral neck BMD, total hip BMD, serum P1NP and serum CTx-I (the dependent variables) and age, BMI, gender, 25(OH)D, *DBP* rs2282679 genotypes, the interaction between 25(OH)D and *DBP* genotypes [25(OH)D × *DBP* rs2282679 genotypes] and fetuin-A. Subjects were then classified into three groups according to *DBP* rs2282679 genotype, and the association between all dependent variables and age, BMI, gender, 25(OH)D and fetuin-A were reassessed by multiple linear regression analysis. The polymorphism of *DBP* rs2282679 genotype were assigned as the following; AA: homozygous referent genotype, CA: heterozygous genotype and CC: homozygous variant genotype. A *p* value less than 0.05 was considered statistically significant. All analyses were performed using the SPSS statistical software package, version 17.0 (SPSS, Chicago IL, USA).

## Results

The mean age of the subjects was 39.9 ± 6.6 years, and most of the subjects were males (72%) due to the demographics of the EGAT workforce. A data comparison between males and females (Table 1) revealed that males were slightly older, and had significantly higher BMI, WC, femoral neck and total hip BMD, serum P1NP and serum CTx-I. With regard to vitamin D status, mean total 25(OH)D concentrations were significantly higher in males than in females (65.20 ± 14.74 vs. 53.64 ± 12.90 nmol/L, p < 0.001; Table 1). As expected, females had a higher prevalence of vitamin D deficiency: 41.4% of females had 25(OH)D less than 50 nmol/L, whereas 13.6% of males were classified as vitamin D deficient. The distribution of 25(OH)D levels in total subject, male and female was shown in Table 2.

**Table 1:** **The clinical characteristics of the study population**

	Total (n = 1,734)	Male ( *n * = 1,246)	Female ( *n * = 488)	*p* value (male vs. female)
Age (years)	39.87 ± 6.65	40.07 ± 6.80	39.35 ± 6.23	<0.01
BW (kg)	66.42 ± 12.93	70.68 ± 11.38	55.55 ± 9.92	<0.001
BMI (kg/m^2^)	23.94 ± 3.76	24.59 ± 3.54	22.27 ± 3.80	<0.001
Waist circumference (cm)	86.11 ± 10.46	89.09 ± 9.42	78.51 ± 9.08	<0.001
Waist–hip ratio	0.88 ± 0.06	0.90 ± 0.05	0.82 ± 0.06	<0.001
Lumbar spine (L1–L4) BMD (g/m^2^)	0.98 ± 0.12	0.98 ± 0.12	0.98 ± 0.10	NS
Femoral neck BMD (g/m^2^)	0.80 ± 0.12	0.82 ± 0.12	0.75 ± 0.10	<0.001
Total hip BMD (g/m^2^)	0.92 ± 0.15	0.95 ± 0.14	0.86 ± 0.15	<0.001
Serum P1NP (ng/mL)	46.28 ± 18.02	48.55 ± 18.48	40.48 ± 15.35	<0.001
Serum CTx-I (ng/mL)	0.35 ± 0.16	0.39 ± 0.16	0.25 ± 0.12	<0.001
Total 25(OH)D (nmol/L)	61.95 ± 15.17	65.20 ± 14.74	53.64 ± 12.90	<0.001
*Subgroup stratified by 25(OH)D levels*				
<50 nmol/L	42.93 ± 5.82 (n = 371)	44.14 ± 4.40 (n = 169)	41.92 ± 6.63 (n = 202)	<0.001
50 to < 75 nmol/L	61.68 ± 6.67 (n = 1,051)	62.28 ± 6.61 (n = 790)	59.87 ± 6.55 (n = 261)	<0.001
≥75 nmol/L	85.45 ± 10.25 (n = 312)	85.64 ± 10.46 (n = 287)	83.26 ± 7.23 (n = 25)	NS
Fetuin-A (μg/mL)	559.16 ± 110.64	560.87 ± 110.29	554.79 ± 111.53	NS

Data is expressed as mean ± SD.

**Figure 1:**
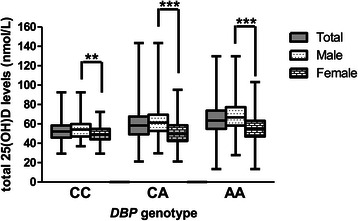
**25(OH)D levels in total subjects, male and female subjects according to*DBP*rs2282679 genotype.** The square box represented mean ± SD. The upper and lower bar represented the upper and lower values. **P < 0.01 for comparison of 25(OH)D levels between male and female in CC group. ***P < 0.001 for comparison of 25(OH)D levels between male and female in CA and AA group.

**Figure 2:**
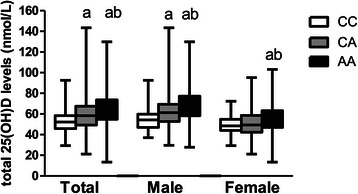
**25(OH)D levels of each*DBP*rs2282679 genotype in total subject, male and female.** The square box represented mean ± SD. The upper and lower bar represented the upper and lower values. **a**: p < 0.05, different from CC group. **b**: p < 0.05, different from CA group.

The *DBP* rs2282679 genotype distribution conformed to the Hardy–Weinberg equilibrium: i.e. AA (999; 57.6%), CA (637; 36.7%), and CC (98; 5.7%). Other than 25(OH)D levels, there were no differences in clinical characteristics between subjects in each *DBP* genotype of the entire cohort (combine males and females; Table 2) or in subgroup of males or females (data not showed). For vitamin D status, subjects with the AA genotype had the highest 25(OH)D levels (64.6 ± 15.5 nmol/L) compared with those in the CA and CC groups (59.2 ± 14.2 and 53.0 ± 10.6 nmol/L, respectively; *p* <0.001, Table 1). For each *DBP* genotype, 25(OH)D levels in males were higher than in females, as presented in Figure 1. 25(OH)D levels in each *DBP* genotype were all significantly different in males (Figure 2). In female, the difference in 25(OH)D levels were found only between those in CC vs. AA and CA vs. AA genotype (Figure 2).

Table 3 shows the associations between BMD, serum P1NP and serum CTx-I, and demographic parameters, 25(OH)D and fetuin-A. Age, gender and BMI were independently associated with BMD at all skeletal sites, as well as serum P1NP and serum CTx-I. 25(OH)D levels were not correlated with any dependent variables, whereas *DBP* genotype tended to be associated with total hip BMD and serum P1NP (*p* = 0.07 and *p* = 0.09, respectively). A weak correlation between fetuin-A and total hip BMD was found (β = −0.05, *p* = 0.02). Since circulating 25(OH)D binds to DBP, it is likely that there is an interaction between 25(OH)D and *DBP* genotypes. The femoral BMD tended to correlate to the interaction between 25(OH)D and *DBP* genotypes *(p* = 0.09). On the other hand, this interaction did not significantly correlate to L1–L4 BMD and bone turnover markers. Subgroup analysis in subject with 25(OH)D < 50 nmol/L (n = 371) did not demonstrate the association between 25(OH)D levels and BMD at all skeletal sites and bone markers (Additional file 1: Table S1).

**Table 2:** **The clinical characteristics of the study population stratified by** ***DBP*** **rs2282679 genotype**

	*DBP* genotype			*P* value
	CC (n = 98)	CA (n = 637)	AA (n = 999)	
M/F (n,%)	69/29 (70.4/29.6)	465/172 (73/27)	712/287 (71.3/28.7)	NS
Age (years)	40.08 ± 6.97	39.96 ± 6.60	39.79 ± 6.66	NS
BW (kg)	66.51 ± 12.09	66.60 ± 13.19	66.30 ± 12.84	NS
BMI (kg/m^2^)	23.67 ± 3.40	23.93 ± 3.82	23.97 ± 3.76	NS
WC (cm.)	86.41 ± 9.90	86.28 ± 10.80	85.98 ± 10.31	NS
WHR	0.88 ± 0.06	0.88 ± 0.07	0.88 ± 0.06	NS
Lumbar spine L1-4 BMD (g/m^2^)	0.98 ± 0.11	0.98 ± 0.12	0.98 ± 0.12	NS
Femoral neck BMD (g/m^2^)	0.80 ± 0.11	0.80 ± 0.12	0.80 ± 0.12	NS
Total hip BMD (g/m^2^)	0.92 ± 0.11	0.92 ± 0.13	0.92 ± 0.16	NS
Serum P1NP (ng/mL)	46.35 ± 15.20	46.22 ± 18.55	46.31 ± 17.95	NS
Serum CTx (ng/mL)	0.36 ± 0.15	0.35 ± 0.16	0.35 ± 0.16	NS
Total 25(OH)D (nmol/L)	53.0 ± 10.6	59.2 ± 14.2	64.6 ± 15.5	<0.001
Fetuin-A (μg/mL)	563.71 ± 112.35	563.01 ± 110.24	556.25 ± 110.75	NS

Data is expressed as mean ± SD.

Because of the potential interactions, data were analyzed according to *DBP* genotype. 25(OH)D levels were significantly related to BMD and bone markers, independently of age, gender and BMI – but only in subjects with the AA genotype (Table 4C). Interestingly, fetuin-A was significantly correlated with total hip BMD (β = −0.09, *p* <0.01), and tended toward a negative correlation with femoral neck BMD (β = −0.05, *p* = 0.08) in subjects with the AA genotype (Table 3C). The correlation effect of both 25(OH)D and fetuin-A was comparable, but in an opposite direction, for total hip BMD, as suggested by the standardized regression coefficients of 0.08 and −0.09, respectively (Table 4C). The result of the correlation between 25(OH)D and BMD and bone markers in subject with vitamin D deficiency was slightly different from the result of the entire cohort. Multiple regression analysis demonstrated the association between 25(OH)D levels and serum P1NP (β = −0.18, *p* = 0.02) in subjects with the CA genotype, independently of age, gender and BMI (Additional file 1: Table S2). In subjects with the AA genotype, 25(OH)D levels were significantly related to L1–L4 BMD, femoral neck BMD and serum CTx (β = 0.30, *p* < 0.001; β = 0.15, *p* = 0.035 and β = -0.15, *p* = 0.03, respectively). On the other hand, the correlation of 25(OH)D levels and total hip BMD and serum P1NP were no longer existed (Additional file 1: Table S2).

**Table 3:** **The association between BMD, serum P1NP, serum CTX and age, BMI, gender, 25(OH)D,** ***DBP*** **rs2282679 genotype, 25(OH)D χ** ***DBP*** **rs2282679 genotype and fetuin-A by multiple regression analysis**

	Lumbar spine L1-L4 BMD		Femoral neck BMD		Total hip BMD		Serum P1NP		Serum CTx-I	
	β	p	β	p	β	p	β	p	β	p
Age	-0.09	<0.001	-0.20	<0.001	-0.12	<0.001	-0.19	<0.001	-0.21	<0.001
BMI	0.22	<0.001	0.39	<0.001	0.37	<0.001	-0.05	0.05	-0.08	<0.001
Male gender	-0.06	0.03	0.13	<0.001	0.17	<0.001	0.25	<0.001	0.44	<0.001
25(OH)D	-0.02	NS	-0.10	NS	-0.11	NS	0.10	NS	0.08	NS
*DBP* rs2282679 genotype	-0.09	NS	-0.14	NS	-0.17	0.07	0.17	0.09	0.13	NS
25(OH)D χ *DBP* rs2282679 genotype	0.11	NS	0.25	0.09	0.26	0.09	-0.25	NS	-0.21	NS
Fetuin-A	-0.02	NS	-0.02	NS	-0.05	0.02	0.04	NS	-0.01	NS

## Discussion

The present findings demonstrated a correlation between femoral BMD and the interaction between vitamin D status, as measured by circulating 25(OH)D, and *DBP* genotypes. It should be noted that statistical interaction does not necessarily reflect true biological interaction. However, a number of biological bases exist which may underlie the observed statistical interaction.

Vitamin D metabolites are mainly transported in the circulation by vitamin D-binding protein (DBP); that is, about 85–90% of 25(OH)D and 1,25(OH)_2_D are bound to DBP. Ten to 15% of vitamin D metabolites circulate weakly bound to albumin, while less than 1% of circulating vitamin D is in free form [22]. Vitamin D bound to DBP is transported within the organism, facilitating access of vitamin D to various tissues and cell types as well as regulating the total amount of vitamin D available for the organism [23]. For example, at the renal proximal tubules, megalin (a cell surface receptor for DBP) internalizes DBP-bound 25(OH)D through endocytosis, and thus free 25(OH)D is further metabolized by renal 1α-hydroxylase [7,24]. In addition, cubilin (another receptor for DBP) and megalin were both detected in osteoblast-like cell lines and human primary osteoblasts cell culture, suggesting that osteoblast are able to internalize DBP-25(OH)D complex *in vivo* [2]. DBP not only acts as a high-affinity serum transporter, but can function as a macrophage-activating factor and actin binder. The direct effect of DBP on bone, independently of its ligands, is not clearly understood. However, most extra-renal tissues do not appear to express megalin or its associated co-receptors, suggesting that these tissues are more likely to acquire 25(OH)D in free form or as bio-available 25(OH)D – i.e. the sum of the free and the albumin-bound fraction of 25(OH)D – and not DBP-bound 25(OH)D [7]. In other words, the free and/or bio-available fraction of 25(OH)D may be more strongly linked to biological effects than the total form. Since only total 25(OH)D is generally assessed in observational and interventional studies, this may explain inconsistencies in the association of total 25(OH)D and health outcomes, including BMD. Supporting this free-hormone hypothesis, some studies have suggested that free or bio-available 25(OH)D, as opposed to total 25(OH)D, is more strongly correlated with BMD [25,26].

Accumulated evidence suggests the influence of *DBP* polymorphism on circulating 25(OH)D. Most studies have investigated three major polymorphic forms of *DBP*: GC1F, GC1S and GC2 (rs7041 and rs4588) [27]. These *DBP* variants exhibit differences in affinity to 25(OH)D and 1,25(OH)_2_D, with the hierarchy of affinity binding GC1F > GC1S > GC2 [8]. Thus, subjects with GC1F alleles had the highest total 25(OH)D levels, while subjects with GC2 had the lowest [28]. Recently, two large genome-wide association studies in populations of European descent reported that rs2282679, another *DBP* polymorphism, had the strongest association with vitamin D deficiency [9,10]. A similar result was reported in two studies of Chinese people [11,12]. That is consistent with the present findings, where total 25(OH)D levels in subjects who had the minor genotype (CC) were about 11 nmol/L lower than in those who had the major genotype (AA). We noticed that the prevalence of *DBP* rs2282679 genotypes in Thais was slightly different than those reported in Chinese [11]: i.e. AA genotype, 57% vs. 45–48%; CA genotype, 37% vs. 42–48%; and CC genotype, 6% vs. 10–11% (in Thais and Chinese, respectively).

Findings from in vitro studies, such as in monocytes [29], dendritic cells [30] and keratinocytes [31], suggest that the biological effects of vitamin D are dependent on both the serum concentration of free 25(OH)D and the *DBP* genotype. For example, monocytes exposed to 25(OH)D showed less induction of antimicrobial cathelicidin in the presence of DBP, while there was much more potent induction of cathelicidin in human cell cultures containing lower-affinity forms of DBP [29]. Similarly, the ability of 25(OH)D to induce dendritic cells to become tolerogenic regulatory T cells was found to be enhanced by either a lower concentration of DBP or by the presence of lower-affinity genetic variants of *DBP* [30].

Based on biological plausibility and statistical interaction between 25(OH)D and *DBP* genotypes, we classified subjects according to *DBP* genotype. Our method differed from previous studies [25,26] in that we did not assess DBP levels directly and did not calculate the amounts of free and bio-available 25(OH)D. Nevertheless, it was found that 25(OH)D was significantly related to BMD and bone markers, independently of age, gender and BMI – but only in subjects with the AA genotype. The analysis in subjects with vitamin D deficiency was mostly corresponded with the result of the entire cohort. Even more the stronger correlation between 25(OH)D levels to L1–L4 BMD, femoral neck BMD and serum CTx were found (Additional file 1: Table S2). We propose that the difference in affinity of vitamin D ligands for DBP and the difference in amount of free and bio-availability forms of 25(OH)D in each *DBP* genotype could underlie our finding. Our study demonstrated that 25(OH)D levels was highest in subjects in AA genotype. Thus, the affinity of vitamin D ligands for DBP is possibly highest in the AA genotype and lowest in the CC genotype. It would be the case that DBP-bound 25(OH)D of subject in AA genotype more reuptake at the proximal tubule, provide more 25(OH)D for renal synthesis of 1,25(OH)_2_D to facilitate circulating levels of this hormone and support endocrine function [27], including bone health. About free hormone hypothesis, a study of Johnsen et al. which explored the effects of rs7041 and rs4588 polymorphisms on BMD, reported that the correlation of the free and bio-available forms of 25(OH)D with bone density were stronger after adjusting for these common polymorphisms [25]. Otherwise, to date there has been little published information concerning the influence of *DBP* rs2282679 polymorphism on BMD. Measurement of DBP levels and additional calculation for DBP-bound/free 25(OH)D in the recent cohort are further warranted to prove this hypothesis. With regard to gender, 25(OH)D of subjects with AA genotype was independently associated with L1-L4 BMD only in females and associated with femoral neck and total hip BMD only in males (data not showed).

**Table 4:** **The association between BMD, serum P1NP, serum CTX and age, BMI, gender, 25(OH)D and fetuin-A by multiple regression analysis in subjects stratified by the** ***DBP*** **rs2282679 genotype**

	Lumbar spine L1-L4 BMD		Femoral neck BMD		Total hip BMD		Serum P1NP		Serum CTx	
	β	p	β	p	β	p	β	p	β	p
A: *DBP genotype = CC (n = 98)*										
Age	-0.13	NS	-0.14	NS	-0.15	NS	-0.25	0.02	-0.20	0.04
BMI	0.23	0.04	0.42	<0.001	0.51	<0.001	0.01	NS	-0.07	NS
Male gender	0.05	NS	0.25	0.01	0.23	0.02	0.08	NS	0.41	<0.001
25(OH)D	0.02	NS	0.01	NS	-0.05	NS	0.05	NS	0.05	NS
Fetuin-A	-0.07	NS	-0.10	NS	-0.08	NS	0.19	0.07	0.16	0.08
B: *DBP* genotype = CA (n = 637)										
Age	-0.09	0.03	-0.21	<0.001	-0.15	<0.001	-0.15	<0.001	-0.19	<0.001
BMI	0.21	<0.001	0.38	<0.001	0.36	<0.001	-0.04	NS	-0.07	0.06
Male gender	-0.03	NS	0.13	<0.01	0.20	<0.01	0.22	<0.001	0.40	<0.001
25(OH)D	0.01	NS	0.02	NS	0.02	NS	-0.01	NS	-0.01	NS
Fetuin-A	0.00	NS	0.04	NS	0.03	NS	0.05	NS	-0.03	NS
C: *DBP* genotype = AA (n = 999)										
Age	-0.08	0.01	-0.19	<0.001	-0.10	<0.01	-0.21	<0.001	-0.23	<0.001
BMI	0.22	<0.001	0.39	<0.001	0.37	<0.001	-0.06	0.07	-0.09	0.003
Male gender	-0.09	0.01	0.13	<0.001	0.15	<0.001	0.28	<0.001	0.46	<0.001
25(OH)D	0.08	0.01	0.10	<0.01	0.08	<0.01	-0.11	0.001	-0.10	0.002
Fetuin-A	-0.04	NS	-0.05	0.08	-0.09	<0.01	0.02	NS	-0.01	NS

Fetuin-A was also demonstrated in the present study to be related to BMD at femoral sites. Fetuin-A is a multifunctional protein mainly of hepatic origin, and plays key roles in calcium and bone metabolism as well as in glucose and energy homeostasis [32]. Fetuin-A is a natural inhibitor of metastatic calcification [32]. However, it has been demonstrated in a number of studies that fetuin-A is positively correlated with bone mass [14,15]. This seemingly contradictory observation has been reconciled by a recent study taking into account the size-selective permeability of collagen fibrils [33]. Although the present study revealed an association between fetuin-A and BMD, the direction of the association was the opposite of previous findings among the elderly [14,15], which demonstrated a positive correlation. One of the differences in the present study which may account for this anomaly is the relatively young age of the study population. Fetuin-A apparently possesses a biphasic response, the underlying basis of which is not entirely clear [34]. The present study also demonstrated that the association of fetuin-A with bone mass varied according to *DBP* genotype, and that this effect was independent of vitamin D status. This observation requires further confirmation, however, and should be taken into account in future studies investigating the effect of fetuin-A on bone.

A number of limitations are present in our study. As mentioned above, our study population was relatively young, with an age range of 25–54 years. Generalizability, if applicable, of our results is therefore limited to this particular age group. The circulating levels of vitamin D-binding protein were not measured, and so we were not able to determine if the interaction between the vitamin D-binding protein gene and vitamin D status was also applicable and could be explained by variations in circulating vitamin D-binding protein. Finally, calcium intake data was not available in the present study.

In conclusion, in young healthy Thai adults, interaction between vitamin D status, as measured by circulating 25(OH)D and *DBP* rs2282679 genotypes, modified the association between total 25(OH)D and bone density and bone turnover markers. Differences in *DBP* genotypes additionally influenced the correlation of fetuin-A levels with femoral sites BMD.

## Additional file

Additional file 1: Table S1. The association between BMD, serum P1NP, serum CTX and age, BMI, gender, 25(OH)D, *DBP* rs2282679 genotype, 25(OH)D χ *DBP* rs2282679 genotype and fetuin-A by multiple regression analysis in subjects with 25(OH)D < 50 nmol/L (n = 371). **Table S2.** The association between BMD, serum P1NP, serum CTX and age, BMI, gender, 25(OH)D and fetuin-A by multiple regression analysis in subjects with 25(OH)D < 50 nmol/L and stratified by the *DBP* rs2282679 genotype.

## Abbreviations

DBP: Vitamin D-binding protein
25(OH)D: 25-hydroxyvitamin D
BMD: Bone mineral density
P1NP: N-terminal propeptides of type 1 procollagen
CTx-I: C-terminal cross-linking telopeptides of type I collagen
EGAT: Electricity Generating Authority of Thailand
WC: Waist circumference
BMI: Body mass index
DXA: Dual-energy X-ray absorptiometry
ISCD: The International Society for Clinical Densitometry
SD: Standard deviation
