# Supplementary material for: Relationship of vitamin D status and bone mass according to vitamin D-binding protein genotypes
Source: Nutr J. 2015 Mar 24;14:29. doi: 10.1186/s12937-015-0016-1 (PMC4389666; doi:10.1186/s12937-015-0016-1)
Supplement: Additional file 1: Table S1. — The association between BMD, serum P1NP, serum CTX and age, BMI, gender, 25(OH)D, DBP rs2282679 genotype, 25(OH)D χ DBP rs2282679 genotype and fetuin-A by multiple regression analysis in subjects with 25(OH)D < 50 nmol/L (n = 371). Table S2. The association between BMD, serum P1NP, serum CTX and age, BMI, gender, 25(OH)D and fetuin-A by multiple regression analysis in subjects with 25(OH)D < 50 nmol/L and stratified by the DBP rs2282679 genotype. [file 12937_2015_16_MOESM1_ESM.doc]

**Supplement data**

**Table 1** The association between BMD, serum P1NP, serum CTX and age, BMI, gender, 25(OH)D, *DBP* rs2282679 genotype, 25(OH)D🗴 *DBP* rs2282679 genotype and fetuin-A by multiple regression analysis in subjects with 25(OH)D < 50 nmol/L (n= 371)

|  | **Lumbar spine L1-L4 BMD** | | **Femoral neck BMD** | | **Total hip BMD** | | **Serum P1NP** | | **Serum CTx-I** | |
| --- | --- | --- | --- | --- | --- | --- | --- | --- | --- | --- |
|  | β | p | β | p | β | p | β | P | β | p |
| Age | -0.08 | NS | -0.19 | <0.001 | -0.10 | 0.03 | -0.12 | 0.02 | -0.11 | 0.02 |
| BMI | 0.20 | <0.001 | 0.40 | <0.001 | 0.37 | <0.001 | -0.04 | NS | -0.10 | 0.05 |
| Male gender | -0.09 | NS | 0.14 | 0.007 | 0.11 | 0.03 | 0.26 | <0.001 | 0.50 | <0.001 |
| 25(OH)D | -0.14 | NS | -0.02 | NS | -0.004 | NS | -0.20 | NS | -0.07 | NS |
| *DBP* rs2282679 genotype | -0.71 | 0.07 | -0.32 | NS | -0.22 | NS | -0.03 | NS | 0.21 | NS |
| 25(OH)D🗴 *DBP* rs2282679 genotype | 0.73 | NS | 0.30 | NS | 0.15 | NS | 0.14 | NS | -0.13 | NS |
| Fetuin-A | -0.01 | NS | -0.05 | NS | -0.11 | 0.02 | -0.05 | NS | 0.01 | NS |

**Table 2** The association between BMD, serum P1NP, serum CTX and age, BMI, gender, 25(OH)D and fetuin-A by multiple regression analysis in subjects with 25(OH)D < 50 nmol/L and stratified by the *DBP* rs2282679 genotype

| **A:** *DBP* genotype = CC (n=37) | | | | | | | | | | |
| --- | --- | --- | --- | --- | --- | --- | --- | --- | --- | --- |
|  | **Lumbar spine L1-L4 BMD** | | **Femoral neck BMD** | | **Total hip BMD** | | **Serum P1NP** | | **Serum CTx** | |
|  | β | p | β | p | β | p | Β | p | β | p |
| Age | -0.24 | NS | -0.36 | 0.04 | -0.31 | 0.07 | 0.01 | NS | -0.02 | NS |
| BMI | 0.10 | NS | 0.43 | 0.02 | 0.47 | 0.01 | -0.02 | NS | -0.01 | NS |
| Male gender | 0.25 | NS | 0.29 | NS | 0.27 | NS | 0.27 | NS | 0.54 | 0.001 |
| 25(OH)D | -0.06 | NS | -0.12 | NS | -0.17 | NS | 0.01 | NS | 0.09 | NS |
| Fetuin-A | -0.20 | NS | -0.19 | NS | -0.01 | NS | 0.01 | NS | 0.28 | 0.06 |
| **B:** *DBP* genotype = CA (n=171) | | | | | | | | | | |
| Age | -0.03 | NS | -0.22 | 0.002 | -0.12 | 0.075 | -0.18 | 0.019 | -0.13 | 0.06 |
| BMI | 0.22 | 0.008 | 0.39 | <0.001 | 0.37 | <0.001 | -0.05 | NS | -0.09 | NS |
| Male gender | -0.13 | NS | 0.16 | 0.036 | 0.24 | 0.002 | 0.23 | 0.005 | 0.46 | <0.001 |
| 25(OH)D | 0.08 | NS | 0.11 | NS | 0.12 | 0.073 | -0.18 | 0.02 | -0.14 | 0.057 |
| Fetuin-A | 0.01 | NS | -0.01 | NS | -0.02 | NS | -0.12 | NS | -0.07 | NS |
| **C:** *DBP* genotype = AA (n=163) | | | | | | | | | | |
| Age | -0.09 | NS | -0.12 | NS | -0.10 | NS | -0.09 | NS | -0.10 | NS |
| BMI | 0.23 | 0.004 | 0.40 | <0.001 | 0.34 | <0.001 | -0.02 | NS | -0.11 | NS |
| Male gender | -0.11 | NS | 0.09 | NS | 0.05 | NS | 0.27 | 0.001 | 0.50 | <0.001 |
| 25(OH)D | 0.30 | <0.001 | 0.15 | 0.035 | 0.06 | NS | -0.13 | NS | -0.15 | 0.03 |
| Fetuin-A | -0.01 | NS | -0.05 | NS | -0.18 | 0.015 | 0.02 | NS | -0.05 | NS |
